# Scope of Iranian community health nurses ‘services from the viewpoint of the managers and nurses: a content analysis study

**DOI:** 10.1186/s12912-022-00908-z

**Published:** 2022-06-08

**Authors:** Aazam Hosseinnejad, Maryam Rassouli, Simin Jahani, Nasrin Elahi, Shahram Molavynejad

**Affiliations:** 1 Student Research Committee, Nursing Care Research Center in Chronic Diseases, Ahvaz Jundishapur University of Medical Sciences, Ahvaz, Iran; 2 Cancer Research Center, Shahid Beheshti University of Medical Sciences, Tehran, Iran; 3 Nursing Care Research Center in Chronic Diseases, Ahvaz Jundishapur University of Medical Sciences, Ahvaz, Iran

**Keywords:** Community health nursing, Task performance and analysis, Iran

## Abstract

**Background:**

Considering the need for nurses specializing in community health and in order to define professional duties for future planning towards creating the position of community health nurses in the primary health care system of Iran, this study aims to explain the range of services which can be provided by community health nurses from the perspective of the mangers and nurses.

**Methods:**

The present qualitative study was conducted with the approach of contract content analysis in Iran in 2020. This study was conducted through in-depth and semi-structured interviews with 22 participants, including community health nursing faculty members, health deputies and managers, community health nurses working in health centers, and the care seekers visiting comprehensive health centers. The samples were selected through purposeful sampling. The interviews continued until data saturation. Data analysis was performed simultaneously with data collection. The interviews were recorded, transcribed, and analyzed through Graneheim & Lundman’s content analysis method, and data management was done using MAXQDA software. To achieve data trustworthiness, the criteria presented by Lincoln and Guba were used.

**Results:**

The obtained data were classified into the two main categories of service provision settings and service provision domains. The category service provision settings covered 7 subcategories including participation in the family physician plan, activities at the centers for vulnerable groups of the community, establishing private community health clinics, leading health promotion programs in the 3rd generation hospitals, activities in comprehensive health centers, follow-ups and home visits, and activities in schools’ health units. Service provision domains consisted of 6 subcategories including participation in health planning, decision-making, and policymaking, research on the health system, health promotion, monitoring and coordination, providing care for the patients with non-communicable diseases and high-risk groups, and eldercare.

**Conclusions:**

From the participants’ perspective, important services that can be provided by the community health nurse are health promotion, the management of chronic patients and the elderly, follow-ups, and home visits. Therefore, it is recommended that health policy makers pay attention to the service provision areas and the services providable by the community health nurse in their macro-planning, and to provide primary health care in comprehensive health centers using inter-professional care models, integrating the community health nurse into the care team.

## Background

The future of nursing will be affected by the vast changes that have taken place in health care. Over the past decade, health care has undergone significant changes regarding technology, the population’s aging, service delivery, the need to manage chronic diseases, increasing public awareness of quality care and cost-effective care. These factors have made the world’s health systems to think about creating opportunities for reform in service delivery [1–3]. Successful change requires a change in the organizational systems of health care provision, especially the nursing profession, because nurses are the largest group of health care providers. As we move towards the future, the provision of nursing services will be transferred from the hospital to other care settings in the community; therefore, in the future, the role of nurses will be different from the current traditional roles [4, 5]. In addition, most people’s first choice for receiving care is home; therefore, the expansion and provision of health services outside the hospital environment by nurses seems necessary [6, 7]. In other words, the role of nurses is very important in supporting the transfer of hospital care to close-to-home centers such as comprehensive health centers. Thus, in order to respond to the needs of the community and provide cost-effective care, nursing services should be used in the community [8].

Community health nursing is a specialty field in nursing that combines nursing science with other health-related sciences (social sciences and public health) and evidence-based practice in various settings in the society, and provides its services to the community with emphasis on improving health, primary prevention, improving social and physical environmental conditions, and rehabilitation. The main approach of this discipline is focusing on community health [9].

Community health nurses in the developed countries of the world play a major role in promoting health. According to the World Health Organization, in these countries, more than 42% of community health nurses work in health centers [9–11]. In Canada, community health nursing has been developed since 1978 and its mission is to maintain and promote the health of individuals, families, and communities [12]. RNs working in primary health care in Canada are divided into two groups: home care nursing and public health nursing. In Canada, Register nurses (RNs) provide services all throughout life (from birth to death) in primary care settings, i.e. they provide services to patients of all ages and are continuously in contact with them. The range of RNs’ services is very wide, including health education, health promotion, prevention, and the management of chronic diseases, acute episodic care, therapeutic interventions, caring for women and infants, and care coordination [13]. In countries like Sweden and Norway, community-based nursing services have been established for several decades [12]. In some European countries, community health nurses have replaced physician-centered and hospital-centered approaches, and provide health services and care to community members [14]. In the United Kingdom and Denmark, community health nurses are hired, in addition to physicians, in the primary health care provision teams and family medical groups. In Germany, community health nurses are used to provide home visit services. In France, community health nurses undergo specialty and specialized training courses to intervene in the community, participate in home visit programs. They put emphasis on promoting health, prevention, and the management of chronic diseases. Among European countries, Finland has the lowest number of physician-patient contacts, and a large number of nurses provide services instead of physicians in the health system [15, 16]. In Ireland, community health nurses operate based on the general model (providing services to patients of all ages, from infancy to old age), and there is ideally one community nurse per 2500 people [17]. In this country, community health nurses are recognized as essential health professionals to support infants and parents, and the first postpartum visit is performed by them with the aim of empowering mothers to care for their infants [18]. In Australia, the community nurse is considered as the primary health care and community-based care provider [17]. A systematic review study by Norful et al. in Spain, New Zealand, the United States, Canada, and Australia summarized a set of roles and responsibilities of RNs in primary care, including vaccination, the assessment of neonatal and infant development, training mothers in breastfeeding and nutrition, performing pap smear, managing chronic patients (hypertension and diabetes), examining diabetic feet, training patients with asthma, following up patients after hospital discharge, prescribing medication, assessing vital signs, triaging patients, ear-washing, maintaining and updating clinical policies and procedures [13].

In Asian countries, community health nursing has not progressed uniformly. In Japan, for example, the role of community health nurses has become more prominent in the last 50 years due to the quadrupling of the elderly population, and they play a key role in implementing health promotion programs [19]. In China, health care services have been assigned to community health nurses since 1997 [14]. In Malaysia, primary health care is highly developed and is provided by nurses in three types of centers: community clinics, maternal and child health clinics, and community polyclinics [20]. In Malaysia, care coordinators’ services are used to manage chronic patients in the clinics providing primary health care, and the nurse is a member of this group. The care coordinator’s roles include following up on clinic attendance, ensuring that patient information is updated, Identifying and tracing visit defaulters and medication refill defaulters, referral tracking, and acting as a bridge between patients and their family health teams [21]. In Oman, community health nursing is not considered as important as hospital nursing and has not been considered by policy makers [22]. In Turkey, community health nurses operate in seven areas: home nursing, maternal and child health and family planning, community-oriented mental health nursing centers, occupational health nursing, and school health nursing. However, in this country, the nursing staff is distributed in a smaller quantity in the provision of health services than in medical and hospital services; 83% of nurses work in hospitals, and only 7% of them work in the centers providing health and family-oriented services [23].

In Iran, the laws of the Ministry of Health, Treatment, and Medical Education and the comprehensive scientific map of the country pay attention to the importance and the role of nurses in promoting health and according to the law of the Sixth Development Plan, the Ministry of Health, Treatment, and Medical Education is obliged to implement “Comprehensive and public health services system” with priority of prevention over treatment, and based on primary health care, with the focus on referral system and family medicine through employing general and family physicians, nursing groups for providing nursing care at community and home levels, service leveling, etc. [4]. Within the framework of Iran’s 2025 Vision Program, it has been mentioned that by 2025, not only should community health nursing graduates be able to meet the changing needs of Iranian society, but they should also have achieved regional and global standards in nursing education and provide effective services to all the members of the society [24]. The existence of such nursing in Iran’s health system, whose method of providing health services is undergoing change, reforming, and moving towards the provision of preventive and community-based services, seems necessary [24, 25]. However, Iranian community health nurses are currently facing obstacles and challenges in providing specialized services in the form of specialized job descriptions developed by the Ministry of Health [12, 26]. According to the statistics of the Ministry of Health, almost all nurses provide services in hospitals and, currently, most health authorities in Iran believe that community health nurses should be employed at the second level of prevention, i.e. clinical care in hospitals [8, 27]. Nurses have not been successful in performing their professional roles due to the changes and the needs of society, and the development of the role of nurses in Iran has not made significant progress [28]. Comprehensive urban health centers in Iran are run by B.S. graduates and associates in the field of family health, environmental health, occupational health, and disease control as well as midwives. In these centers, health services are provided sporadically [14] and the expertise of community health nurses is not used in care provision to provide services in accordance with the needs of the community [29, 30]. Therefore, considering that community health nurses have been trained through a holistic approach, they pay attention to all the aspects of personal health and the social factors affecting health in planning and providing care. Besides, in the phase of planning and implementing the interventions, they take into account the socio-economic status of the care-recipient and his/her family in order to design a care program tailored to his needs [31]. Community health nurses are one of the key members of the health team who identify major environmental and health problems of the community and also design, plan and implement the necessary health measures in cooperation with other team members, by using the nursing process in various fields of the community. They also have sufficient skills, in addition to clinical and managerial knowledge, to lead and manage care, and have key competencies to coordinate the care team and manage patients’ transfer from medical and hospital centers to comprehensive health centers and follow them at the community level [32]. On the other hand, community health nurses have the ability to participate in health policy-making as a consultant, and play a key role in regional crisis management [33]. Therefore, the above-mentioned roles that are not performed in comprehensive health centers can be assigned to community health nurses.

Since the integration of nurses in the primary health care team with the aim of supporting high quality care is necessary and, on the other hand, as the lack of a clear definition of the role of community health nurses leads to not using them properly and denying them the opportunity to participate in providing quality care, it is necessary to determine the range of the services that can be provided by community health nurses in Iran’s health system. Determining community health nurses’ role in the health system requires considering the beneficiaries’ opinions. Therefore, a qualitative research is the best way to investigate different perspectives, because values and social, cultural and human aspects cannot be fully studied through quantitative approaches [34]. In addition, qualitative studies can be conducted for in-depth description of unknown or less-known phenomenon from the perspective of those who have experienced it [35]. Thus, the present study is carried out aiming to explain the scope of community health nursing services, according to nurses and mangers’ opinions.

## Methods

### Aim

The aim of the study was to describe the range of services which can be provided by community health nurses from the perspective of the stakeholders.

### Design

In this qualitative study, the conventional content analysis approach was used. This study complied with the consolidated criteria for reporting qualitative research (COREQ) checklist [36].

### Setting and participants

The study participants included the faculty members of community health nursing, the managers and the health deputies of medical universities, the community health nurses working in health centers, and the care-seekers referring to comprehensive health centers, who were selected through purposive sampling method. The inclusion criteria were having at least 10 years of work experience in an educational environment or 5 years in health centers, and the willingness to participate in the study. Regarding the care-seekers, having an electronic file in a comprehensive health center was necessary. A total of 22 participants were interviewed until the data was saturated. The research objectives were explained to the participants. Their voluntary participation and data confidentiality were ensured. After obtaining the written informed consent, the date and the place of interviews were set by the participants.

### Data collection

The study data were collected through in-depth, semi-structured interviews from May 2020 to February 2021. After obtaining the permission to conduct the research from the Vice Chancellor of Research of Ahvaz Jundishapur University of Medical Sciences, eligible samples were selected. Due to the specific circumstances of COVID-19 epidemic, 7 interviews were conducted via Skype or WhatsApp. The rest of the interviews were conducted face to face. To maintain the result’s credibility, a test interview was performed where the questions were tested. The test interview was not included in the study. The consent forms were sent to the participants by email and they sent the filled form back before the interview. All the interviews were audio recorded with the consent of the participants. The interview questions included “Based on your experience, what is the range of the services that nurses are able to provide?”, “Based on your experience, what are the service gaps in the network system, and how can nurses be used in the network system to fill these gaps? “, “As a community health nurse, which roles and responsibilities do you undertake in promoting community health and towards achieving the goals of your discipline?”, “Which services would you like to be provided with in comprehensive health centers?” The interviews were then followed by exploratory questions such as “Could you give more explanation?” and “Could you give an example?”. Each interview lasted between 30 and 60 minutes (mean: 45 min). Totally, 22 interviews were conducted. Only the first author and the relevant participant were present at each interview. The interviews were transcribed, including notes regarding body language and tone of voice in order to facilitate the interpretation of the result.

### Data analysis

Data analysis was performed simultaneously with data collection. In order to begin data analysis, the recorded interviews were listened to several times and written by the first researcher. The first and the second authors performed the analysis with the help of the other three authors. Data management was performed using MAXQDA-v10 software, and qualitative data analysis was done based on the proposed five-step method of Graneheim and Lundman. These steps include writing the whole interview immediately after each interview, reading the full text of the interview to get a general understanding of its content, determining the meaning units and basic codes, classifying similar primary codes in more comprehensive classes, determining the hidden content in the data and extracting the main theme [37]. For this purpose, the interviews were written immediately after completion. Then the transcripts of the interviews were reviewed several times for general understanding. Since qualitative research requires immersing in data, the researcher listened to the interviews several times. An abstract was then written for each interview, and the key sentences were underlined and its overt and covert contents were extracted. Then the meaning units were extracted from the sentences used by the participants in the form of initial codes (*n* = 233). These codes were classified based on semantic and conceptual similarities. It should be noted that the data reduction process took place in all the analysis units as well as the main categories and the sub-categories. An example of data analysis is reported in Table 1.

**Table 1:** Detailed information about the main categories and the subcategories

Quotation	Basic codes	Subcategories	Main categories
“If I were a nursing leader, I would definitely link nursing to economics. The nursing institution in general and the community health nursing institution in particular talents must enter this discipline. In my opinion, leadership should be able to create a good financial value for the community health nurse. I have repeatedly said that we are not a university of public administration must have a good professional future for the ones who enter it, and the best for the production of government employees. We want to train entrepreneurs.”	Entrepreneurship for the community health nurse The need to create added value for nursing services in the community	Establishing private clinics by a community health nurse	service provision settings
“A large percentage of people in the community need care, training, and counseling due to chronic illnesses, and a community health nurse can provide them with self-care and self-management training as well as necessary follow-ups, in person or even virtually due to COVID-19 conditions.”	Managing the complications of non-communicable diseases Teaching self-care and self-management to patients and their families	Providing care for the patients with non-communicable diseases and high-risk groups	service provision domains

### Rigor and trustworthiness

Lincoln & Guba’s criteria were used to confirm the trustworthiness and the credibility of the study [38]. The credibility of the study was assessed through the researcher’s continuous engagement with the research data for 10 months, returning the texts of the interviews to the participants, and obtaining their approval, and reviewing the interview texts, codes, and themes by three experts and supervisors. The dependability was achieved by combining several methods of data collection including interview, observation, and note-taking. In addition, the data was reviewed by an outside observer familiar with qualitative research. In order to achieve data confirmability, all the stages of the research, especially data collection, data analysis, and categorization, were recorded in detail in order to provide the possibility of auditing the research by the audience and the readers. Finally, the characteristics and the experiences of the participants were well described in order to achieve the criterion of transferability. In addition, the participants were also selected with maximum diversity.

## Results

The study was conducted on 22 participants consisting of 10 males and 12 females, in 4 groups. Finally, 8 community health nursing faculty members affiliated to medical universities (mean age = 50.50 ± 4.72 years), 7 health managers, and deputies from medical universities (the mean age = 51.71 ± 9.53), 4 community health nurses working in health centers (the mean age = 39.67 ± 4.72 years) with a working experience of 12 to 30 years (the mean value = 21.8 years) and 3 care-seekers visiting comprehensive health centers with a mean age of 41.67 ± 8.62 years participated in the study. The codes extracted from the interviews led to the formation of two main categories: “service provision settings” and “service provision domains. The information about the main categories and the sub-categories is reported in Table 2.

**Table 2:** The main categories and the sub-categories extracted through conventional content analysis of interviews

Main categories	Subcategories
**Service provision settings**	Participation in the family physician plan
	Activities in the centers for vulnerable groups of the community
	Establishing private community health clinics
	Leading health promotion programs in the 3rd generation hospitals
	Activities in comprehensive health centers
	Follow-ups and home visits
	Activities in school health units
**Service provision domains**	Participation in health planning, decision making, and policy making
	Health system research
	Health Promotion
	Monitoring and coordination
	Providing care for the patients with non-communicable diseases and high-risk groups
	Elderly care

### A) Service Provision Settings

One of the main categories is community health nursing service provision settings. Service provision settings are the settings where community health nursing services can be provided. The information regarding service provision settings is divided into 7 subcategories: “Participation in the family doctor plan”, “Activities in the centers related to vulnerable groups in the community”, “Establishing private community health clinics”, “Leading health promotion programs in the 3rd generation hospitals”, “Activities in comprehensive health centers”, “Follow-ups and home visits” and “Activities in the school health unit”.

#### 1. Participation in the family physician plan

The family physician program is one of the settings mentioned by the participants as one of the community health nurses’ service provision settings. Regarding family medicine, there is a requirement for a person to work along with the physician as a health care provider. Since there are no graduates called health care provider, this role has been assigned to midwifery and family health experts. Thanks to the expertise, abilities, and skills of community health nurses in working with people at the community compared to an expert, they can fulfil this role.

“In the family physician program, either, they have not considered a role for the nurse, because they use midwives as multipurpose manpower, which means that they believe midwives can do several things at the same time (child and mother care, and family planning). However, no position has been created for nurses, and they did not take them into account, and the health care assistants of family physicians who are supposed to be nurses are replaced by midwives and the health care experts holding associate degrees. I attended one of their meetings and told the then-president of the university that we have a discipline called community health nursing, which is known in the world. Let’s enhance this discipline and include community health nurses in the family doctor plan,” one of the faculty members of community health nursing said on the matter.

#### 2. Activities at the centers for vulnerable groups of the community

The participants referred to the skills of community health nurses to work and provide care for care seekers in the police force, prisons, care centers for the intellectually disabled, day care and permanent care centers for the elderly, the educational system, counseling centers for behavioral disorders, cooperation with the Welfare Organization and Imam Khomeini Relief Foundation.

“The Welfare Organization, the Ministry of Education, and the police force also work with groups that are part of our vulnerable community. They deal with all kinds of care-seekers, nursing homes, the disabled, incarcerated care-seekers and their families, and students, but they do not look at this issue from the perspective of community health. Such centers are among the best places for community health nurses to work.” Said a provincial health deputy stated in this regard.

#### 3. Establishment of private community health clinics

The participants emphasized the need to give community health nurses the freedom to operate in the private sector through establishing clinics and community health complexes. They stated that for this purpose, the job description of community health nurses should be determined in the centers and tariffs should be set for their services. Then licenses should be given to community health nurses who have acquired professional qualifications.

“Apart from the network, we must activate nursing agents, i.e. nursing homes and community health nursing specialty clinics, in the treatment system. Since the health network system is governmental, it is the government’s responsibility to provide it to the public for free, but all governments need to assign the additional services to the private centers to create a healthy competition, or in order for people to be able to receive services privately as well,” one of the faculty members of community health nursing stated in this regard, “In the system of nursing service provision, these services should be provided through home-based or community-based nursing care centers. Currently, we only have nursing counseling and home-based care centers that only have to do with treatment and do not have any special intervention in the field of health promotion, prevention, and education. But if nurses can have community health clinics in the community, which are directed by community health nurses, especially for non-communicable diseases, as the cost of nurses’ services is definitely different from that of a specialist, people can refer to such clinics at a lower cost.”

#### 4. Leading a health promotion program in the 3rd generation hospitals

The participants emphasized community health nurses’ ability to lead the health promotion programs of the 3rd generation hospitals.

“Third-generation hospitals look at the health of the region in which they are located. If they see, for example, that most of their clients are diabetics who are being amputated, they design and implement programs in order to prevent diabetic foot. Basically, we should have this program in health care. Hospitals identify the needs of their regions and take measures towards the health of the region in order to reduce the burden of their referrals and to receive patients in better conditions. Therefore, in my opinion, community health nurses can happen to have a very important role in coordinating and directing health programs in these hospitals. Since community health nurses also have the knowledge of epidemiology, they analyze the community and its information, and design the necessary intervention.” Now, the important issue in hospitals is the promotion of hospital health, the 3rd generation hospitals pay attention to the health of the people in the region where they are located. In these hospitals, not only are they responsible for treatment, but it is also important to promote the health and prevent diseases in the people who are under their coverage. These hospitals are an area of activity for community health nursing. The World Health Organization also has a unit called Health Promoting Hospitals, which started operating in 2006 and have international networks which can be connected through the activities of community health nurses,” an executive health deputy of one of the provinces said in this regard. (Female, Community Health Nursing faculty member, 28 years of work experience).

#### 5. Activities in comprehensive health centers

All the study participants believed that one of the important settings of community health nurses’ activity are comprehensive urban and rural health centers. According to the participants, community health nurses along with the center’s physician can work as community-oriented nurses and communicate well with people. The community health nurse must be present in the heart of the community and work with people and for people. The nurse who is in the hospital works in a completely closed environment and has no contact with people, but the nurse who works as a health care provider is in daily contact with people.

“With the capabilities defined for a community nurse, they can be located in rural and urban health centers, whether in the public or the private sector, and given that there already are some forces in these centers, we can add community nurses, or, in some places, they can replace the existing forces, for example, midwives can be transferred to the reproductive health field, and the community nurse can replace them in that unit,” said one of the health policy makers of the Deputy of Health on the matter.

#### 6. Follow-ups and home visits

Most of the participants stated that one of the community health nurses’ settings of service is following up the patients at the community level and perform home visits. Currently, the health system does not perform special interventions for the patients discharged from the hospital, while the patient and his/her family usually have a lot of questions regarding care after discharge. Moreover, due to the high number of accidents in the country, there are many people with spinal problems who need to be given care at home. On the other hand, there is a need for regular follow-ups and trainings in chronic diseases. Besides, COVID- 19 disease showed the need to provide care and follow-ups to the patients with mild to moderate levels of the disease in the community. The participants believed that since community health nurses have learned the theory of home visiting, they can interact with the community and the family, perform home visits, write nursing diagnoses, and perform necessary interventions for them. In the current situation, due to COVID-19 pandemic, community health nurses can perform training and follow-ups through home visiting virtual networks, i.e. They can design a system for people to ask their questions and receive the answers without the need for in person referrals.

“The most important principle the absence of which is felt in our centers is the discussion of the follow-ups that need to be done, especially in the case of chronic diseases, cancers, and the elderly. On the one hand, the workload of health care providers is high, on the other hand, they do not specialize in some aspects of this care, but if we have one person to do these follow-ups professionally in each region according to the population, it will solve our problem as well,” said a provincial health deputy in this regard.

“At my own level, I did what I could, even though we did not have a law to support home visit. During my 28 years of work, I performed more than 100 home visits, which were helpful (for patients with high blood pressure, diabetes, tuberculosis, disabled children, etc.) But it was all informal, based on relationships. I mean I could not refer these people to the hospital, for example, Imam Hossein Hospital, with a note issued by the health center. My students themselves contacted the students who were in the hospital and made an appointment to refer the family. While the right way of doing it is through the network system.” (Female, Community Health Nursing faculty member, 28 years of work experience).

#### 7. Activities in school health units

One of the community health nurses’ service settings pointed out by the participants was working in school health units.

“One of the settings that we easily have access to for the activities of community health nurses is schools, which do not need either special infrastructure or physical resources, because community health nurses have the necessary knowledge and skills to promote health and prevention at different levels and can provide the required health services to the three groups of students, instructors, and parents,” one of the faculty members of community health nursing stated in this regard.

### B) Service Provision Domains

The study participants pointed to the service provision fields that can be defined for the community health nurse, which do not overlap with the roles of other health care providers. This category has 6 sub-categories including *participation in health planning, decision-making, and policymaking*, *research on the health system*, *health promotion*, monitoring and coordination, *providing care for the patients with non-communicable diseases and high-risk groups*, and *eldercare*.

#### 1. Participation in health planning, decision making, and policymaking

In this study, the participants stated that one of the service provision domains that can be assigned to community health nurses in comprehensive health centers and bases is the identification of the health needs of the people in the region, and planning and decision-making for different groups of people (schools, families, etc.) in that region.

“If we want to improve the position of nurses in community health, they should be entrusted with policy making and planning in the regional health care, which is sometimes known as family medicine. Then we tell the community health nurse that he/she is responsible for a population of, let’s say, 4,000 people, that we leave them to him/her with all their sexual, age-related, and social class characteristics, and that he/she should have a plan for them. A competition must be created. For example, how much have the indicators improved in the region for which Ms. x is responsible compared to another area. Therefore, I think that if we want to guarantee the health of our community, this guarantee of health services must be entrusted to competent people at all levels, and the best person who has this competence is the community health nurse,” one of the faculty members of community health nursing said in this regard.

#### 2. Health system research

One of the fields considered for community health nurses is research. They should cooperate and participate in conducting and developing health-based and population-based research in the health system, and use the research results to develop and improve the quality of health services. The participants mentioned this issue, too. In this regard, one of the faculty members of community health nursing mentioned:

“Considering that the community health nurse has learned to be a good observer and has passed a research methodology course, too, he/she can find some health issues through detailed observation in the comprehensive health centers, work on them using the research methods that he/she knows, and report the results to higher level authorities. A health care provider does not do this job because he/she doesn’t have such a point of view at all. A health care provider just wants to do his/her job in the center and leave. That’s all. In other words, research tasks can be defined for community health nurses so that they will become sensitive to community health issues.”

#### 3. Health promotion

From participants’ perspective, one of the service provision domains of community health nursing is health promotion and prevention services.

“In my opinion, if we increase the level of education at the community, i.e. if community health nurses work in the field of health promotion, they can affect the level of education among all groups of people at the community. This itself is a first-level prevention and will reduce the care burden of hospitals,” one of the community health nursing faculty members stated in this regard.

#### 4. Monitoring and coordination

In team work, community health nurses play a good role in monitoring and making coordination. Participants believed that community health nurses could take responsibility for comprehensive health centers and facilities and monitor the performance of other executive forces, including health care providers and behvarzes (rural health care workers).

“I think a good observer is someone who knows the whole processes and the programs, and is scientifically one level higher than health care, someone who is at least a master’s graduate, knows diseases and their physiopathology, knows epidemiology. This person can evaluate the performance of staff such as health care workers and bahvarzes according to the protocols communicated by the County’s health center, is able to give feedback and intervene to improve their performance. I think that a community health nurse can help in this regard because working with the community is very important and a nurse is able to do so,” said the director of communicable diseases department of a provincial health deputy.

#### 5. Care provision for patients with non-communicable diseases and high-risk groups

One of the domains of community health nursing services that participants focus on was the care provision and follow-up of patients with non-communicable diseases.

“Based on my experience in this unit, I think that for non-communicable diseases, which especially now account for 76% of the disease burden, that we want to consider individual care and interventions, we may not be able to achieve control and prevention goals very soon. To achieve this, we must have a socialist approach in order to be able to control these diseases, because many of the risk factors for diseases are factors that are not directly under the control of the Ministry of Health, in other words, they are social determinants. Let’s plan carefully. In my opinion, we now have individual-level care by health workers, but in order for our approach to non-communicable diseases to be community-oriented, we need a series of capable people at a higher level, such as community health nurses who are stationed in comprehensive health centers,” said the director of non-communicable disease department of the health deputy of one of the provinces.

#### 6. Elderly care

Our society is moving towards aging, specifically by 2035, the number of elderly people will increase to a great extent, but comprehensive services to this group have been neglected. Therefore, one of the priorities is the health of the elderly. Community health nurses can provide them with comprehensive services in the field of prevention and eldercare thanks to their knowledge of different groups, including the elderly. In this regard, one of the care-seekers visiting the Comprehensive Health Center stated, “The health experts in these centers cannot give me good advice about the drugs I take. I even once asked them about how to inject my insulin and its side effects. They had no idea. They only measure my blood pressure and weight, which I can do at home by myself using gauges. Maybe if there were a nurse in this health center, like when I am hospitalized, before being discharged, he/she could give me better explanation about my medication.”

## Discussion

The scope of services, the definition of the role, and the description of the duties of community health nurses are somewhat different in different communities. Factors such as the laws governing the community health system, the structure of the health system, the needs of communities and the needs of care-seekers affect the description of community health nurses’ duties [39]. In other words, the scope of services provided by community health nurses in a country is usually dependent on the culture of the community and the range of services provided in the health system [40, 41]. This study was designed due to the need of Iran to provide services by nurses specializing in community health and in order to explain the scope of these nurses’ services from the perspective of the stakeholders. The results of this study led to the identification of two main categories: “service delivery settings” and “service delivery domains”.

The findings showed that the settings in which community health nurses can provide their services include “Participation in the family physician plan”, “Activities in the centers related to vulnerable groups in the community”, “Establishing private community health clinics”,“ Leading health promotion programs in the 3rd generation hospitals”, “Activities in comprehensive health centers”,“ Follow-ups and home visits “, and“ Activities in school health units”.

In the present study, participation in the family physician plan was mentioned as one of the important settings of community health nurses’ activities. Askari’s study points to the importance of the presence of nurses in the family physician team, but the role of nurses in the family physician team is not precisely defined. Besides, in the executive instructions of this project, the term “a nurse or a midwife” has been used to define the role and the position [42]. Although the family physician project has been started in Iran, it is not enough, and both the government and the people will need to pay its costs. A community health nurse can be a complementary project to that of the family physician and even make up for its deficiencies. However, in the first step, the role of the community health nurse in the structure of health care systems must be defined. This can help the government in perceiving the goal of “health for all”. As the presence of nurses in blood pressure screening in 2012 confirms this [43].

The participants mentioned “working with vulnerable groups in the community” as one of the settings of community health nurses’ activities is. In this regard, a study conducted by the World Health Organization has pointed out community health nurses’ ability to deal with the care-seekers who belong to vulnerable groups, such as single-parent families, the families with drug-abusing fathers, the families with a member suffering from AIDS, and the families with a child who has become disabled following a car accident [9]. In his study, Ahmadi has mentioned that it is necessary for the health care provider system to adapt to the needs of single-parent families (widowed women who are heads of the family) in order to monitor these women’s health and improve their quality of life. He has also referred to the necessity of defining community health nurses in the health system, as they are able to provide health and support services in accordance with the needs of this group [44].

A study examining the involvement of community health nurses in primary health care in the UK found that this country possesses one of the models of community health nurse activities, in which nurses work independently in clinics and are responsible for managing the clinic and providing care, training, and counseling, and even have the competence for drug administration [45]. In another study, Maier examined the transfer of duties from physicians to nurses in primary health care in 39 countries, and pointed out the transfer of duties to nurses in more than two-thirds of these countries. She also found that in 11 countries under study, the job of visiting patients has also been assigned to nurses. She states that since most countries invest in the training of specialist nurses, employing them requires the creation of a flexible health structure and the provision of resources [46]. In this study, too, the participants stated that community health nurses should be entrepreneurs in order to have activities besides governmental sector and centers, and must establish private community health clinics with the aim of providing health promotion, prevention at various levels, and counseling services. In his study, Shirjang considers ignoring private sector in the structure of primary health care as one of the challenges of primary health care and pointed out the need to design an appropriate outsourcing model in order to use the capacity of the private sector in providing primary health care services [29].

The goal of health-promoting hospitals is to transform a hospital from a mere place of diagnosis and treatment to a place of disease prevention and health-promotion for patients, staff, clients, and all the members of society. The mission of this type of hospital is to change the “treatment-oriented attitude” to “health-oriented attitude” [47]. In our country, the role of prevention is almost exclusively the responsibility of environmental levels in the health network system, and medical centers play the same traditional roles of diagnosis and treatment, and there is no specific program to promote health in hospitals. That is why it is necessary to define new health promotion services for hospitals [48]. Therefore, considering the important role of community health nurses in health promotion, they can play a significant role in hospital health promotion services [49]. In the present study, the participants mentioned that one of the important settings of community health nurses’ activities is the hospital, with the aim of turning hospitals into health promotion hospitals.

One of the designated positions in the curriculum syllabus of Community Health Master’s program is health centers. All the study participants also believed that one of the settings of community health nurses’ activities is comprehensive health centers. A study aimed at strengthening health systems through nursing in 14 European countries found that there are health centers in Sweden and Finland where community health nurses are the first to be in contact with care-seekers. In these countries, primary health care is provided by community health nurses, and they provide a wide range of services in the medical, health, and social domains in the form of health promotion, prevention, diagnosis, screening, treatment, palliative, and rehabilitation activities for people [11].

In the present study, following up patients in the community and home visits have been mentioned as one of the important settings of community health nurse activities, with the aim of better management and control of diseases and reducing treatment costs. In this regard, the study of Landers, which examined the future of home health care, highlighted the importance of the role of following up and home care by the nurse in improving the patient’s experience of care (improving the quality of care and patient satisfaction), promoting community health, and reducing costs [50]. In addition, in a study, Konlan showed that one of the main activities of a community health nurses in Ghana is home visiting. Ghana’s health system supports basic community health interventions through home visits and addresses the gaps in community knowledge and practice (such as the reproductive behavior, women and children’s nutrition, early diagnosis, disease prevention, and patient management at home [51]. The study of Barrett also showed that one of the most important settings of community health care nurses’ activities is the home environment, because the care-seeker has more power, control, and influence there; therefore, they will participate more in their training and care programs. In addition, other family members who are involved in decision-making regarding training and care programs will be present through home visits [52]. In Norway, too, community health nurses specifically provide health and care services to the mothers who have recently given birth and their infants, and the elderly in the region through the home visit program. The outcome of such programs is early discharge from the hospital, the reduction of hospital workload, and the empowerment of care-seekers in self-care [53]. In a review study, Karlsson pointed out the impact of home visit programs on reducing unnecessary emergency visits by the elderly and the development of elderly self-care [54].

The health of children and adolescents is multidimensional and requires the cooperation of various professions, including community health nurses. In today’s health system, it is very important to pay attention to cost-effectiveness. Employing community health nurses to provide school health services is recognized as an effective strategy in reducing health system costs [55]. In the present study, one of the settings of community health nurses’ activities is the school health unit. Community health nurses play a significant role in coordinating and providing public health interventions for school children. The study of Hoekstra also noted the diverse roles of nurses at schools. One of the most important roles of nurses at schools is providing health education and counseling. Two groups of important interventions that can be provided by community health nurses at schools are reducing high-risk behaviors and promoting self-care [56].

The findings showed that the domains of service delivery which can be taken on by community health nurses include “participation in health planning, decision making, and policy making”, “health system research”, “Health promotion”, “Monitoring and coordination”, “Providing care for the patients with non-communicable diseases and high-risk groups”, and “Eldercare”.

According to the Community Health Nurses of Canada, community health nurses should use decision-making strategies such as the nursing process, which combines judgment, practice, responsibility, and accountability. Community health nurses identify the needs of the region, plan, and make the necessary policies to meet the needs, and implement and evaluate those policies with the participation of other health care providers and other law enforcement agents in the region [33]. In the present study, the participants considered planning, decision-making, and health policy-making as one of the domains of providing community health care services. They believe that most of the physicians who direct the centers do not have any knowledge of the community covered and their health needs for decision-making and policy-making, since most of them are passing their compulsory medical service program. Besides, in some centers, where a midwifery or health expert is in charge of the base or the center, unfortunately, they do not have comprehensive scientific information regarding health policy making and planning. The study of Shirjang also showed that physicians working in health centers only do the treatment job and do not have the ability to play a role in prevention and care programs [29] because their university education is not in line with the new needs of the society and PHC [30].

The participants believed that community health nurses in comprehensive health centers should conduct research based on the problems in the region and use the results of their research for evidence-based decision making. According to the Community Health Nurses of Canada, community health nurses identify and support the research on key community health issues and approaches, and participate in research projects. If possible, they use collaborative research methods to involve community members in planning or conducting research, and share research date and evaluation information with colleagues, instructors, nursing students, other professionals, and the general public [33]. In a study, Barthow showed that there is a lot of evidence to support the research nurse in the hospital and the clinical environment, while the role of the research nurse in community-based research is less known [57].

Nurses have a unique position to work in the field of health promotion. Nurses’ actions in this field include disease prevention and health education, which are done with the participation of other health team members [58]. This is especially the case for community health nurses who work in community-based settings, prisons, schools, health-promoting hospitals, and planning and management situations where there is ample opportunity for health promotion [49]. In the present study, health promotion was mentioned as one of community health nurses’ service provision domains. The World Health Organization also considers health promotion as one of the main interventions of community health nurses in the primary health care system [9]. All around the world, the focus of the health system has shifted from patient-centeredness to providing health promotion services [59].

According to the participants, one of the domains of service provision that can be assigned to community health nurses in health bases and comprehensive health centers is monitoring other health care providers and coordinating activities, which do not overlap with the roles of other health care providers. In the study of Swanson, coordinating care among health team members was mentioned as one of the key roles of the nurse in primary health care [59]. According to the World Health Organization, community health nurses actively monitor the health activities and processes in the region and provide decision makers and community members with monitoring data in an understandable form. Community health nurses use intermediary skills to facilitate inter-organizational and intra-departmental collaboration. They also use cooperation and participation towards promoting and protecting health [9].

Due to the increase in the number of the patients with chronic diseases and the need for counseling, primary health care, and proper follow-up, it is necessary to use the capabilities of nurses in the community. In a study, Yousefi also pointed out the importance of nursing counseling in better management of the patients with chronic diseases, cost-effective care, improving health care outcomes, and promoting the health of the patients with chronic diseases [60]. The participants in the present study also pointed out the management of non-communicable diseases as one of the important duties of the community health nurses. The results of Crespo model in the management of chronic diseases are also in line with this study. The outcomes of using this model for the patients with diabetes include disease management through controlling the blood sugar level and reducing HbA1C in a short time, coordination between different types of care, patient satisfaction, and reducing costs [61]. In a systematic review study, Norful showed that in the United States, Australia, Canada, New Zealand, Spain, and South Africa, nurses play a major role in the management of chronic patients. Blood pressure and diabetes were two common chronic diseases, and the nurses were responsible for evaluating, controlling, and following up these patients [62]. Karlsson’s study showed that the clients with chronic illnesses were satisfied with nursing counseling. The clients were less inclined to visit a doctor frequently, for they needed to spend more time and money [54].

Eldercare was mentioned as one of the domains of community health nurses’ service delivery in the present study. The participants believed that nurses, compared to other health care providers, had the necessary scientific and practical competence to instruct, counsel, and provide care for the elderly in comprehensive health centers. In Sweden, nurses act as health gatekeepers to promote health and prevent diseases and play a key role in providing care to the elderly [63]. In a study, Kabayama also pointed out the importance of community health nurses in providing long-term services to the elderly in care centers. In addition, in order to have a healthy elderly community, the demand for health promotion services provided by community health nurses is increasing [19].

## Limitations

The COVID-19 pandemic has made it difficult for face-to-face interviews, due to the long distance between the researchers and some professors, specialists, and experts in the field of health in the country. This restriction was largely removed with the professors’ cooperation, through conducting some interviews on Skype and WhatsApp.

## Conclusions

Based on the results of this study, important services that can be provided by community health nurses include health promotion, the management of chronic patients and the elderly, follow-ups, and home visits. Due to the fact that non-communicable diseases impose additional financial burden on individuals and the government, it is recommended that health policy makers, in their macro-planning, consider the services provided by the community health nurse, which do not overlap with the duties of other health care workers in the network system. In addition, it is recommended that, in order to provide primary health care in comprehensive health centers, health policy makers use inter-professional care models, integrating the community health nurse into the care team. Determining the range of national services providable by community health nurses may help transform the health system by improving the performance of the health care team, supporting inter-professional performance, and maximizing the scope of nursing services.

## Data Availability

The datasets used and/or analyzed during the current study are available from the corresponding author on request.

## Abbreviations

PHC: Primary Health Care
COREQ: Consolidated criteria for reporting qualitative research
